# The Association Between Frequent Sugar-Sweetened Beverage Intake and Sleep Duration in School Children: A Cross-Sectional Study

**DOI:** 10.3389/fnut.2022.847704

**Published:** 2022-03-15

**Authors:** Ya-Hui Shih, Hsin-Chuan Wu, Wen-Harn Pan, Hsing-Yi Chang

**Affiliations:** ^1^Institute of Population Health Sciences, National Health Research Institutes, Miaoli County, Taiwan; ^2^Department of Food Nutrition, College of Human Science and Technology, Chung Hwa University of Medical Technology, Tainan City, Taiwan; ^3^Institute of Public Health, National Yang-Ming Chiao Tung University, Taipei, Taiwan; ^4^Institute of Biomedical Sciences, Academia Sinica, Taipei, Taiwan

**Keywords:** sugar-sweetened beverages, sleep duration, sleep debt, FFQ food frequency questionnaire, IPAQ

## Abstract

**Background:**

Higher consumption of sugar-sweetened beverages (SSBs) maybe association with children's sleep pattern. However, few studies have considered this association in Asia, especially in school children. This study investigated the relationship between children's consumption frequency of such beverages and their sleep duration.

**Methods:**

Participants aged 6–12 years were analyzed from two survey data in 2012 and 2013–2016 Nutrition and Health Surveys in Taiwan. A total of 2,628 participants were included in the analysis (2012, *N* = 1,267; 2013–2016, N = 1,361). Beverages weekly consumption were divided into low and high intake groups by medians cut-off points. The sleep variables were the sleep duration at night (including school days and weekends) and sleep debt. After controlling the confounders, the correlation between sugar-sweetened beverage consumption and sleep duration was examined using multinomial logistic regression analysis.

**Results:**

The students slept for an average of 8.8 h on school days and 9.7 h on weekends. Relative to the low SSB intake group, the high intake group exhibited shorter sleep durations on school days (*P* < 0.001), greater sleep debt (*P* = 0.049). In logistic regression, high intake group were more likely to sleep for <8.5 h on school days (OR = 1.67, *P* = 0.002) and exhibit >2 h of sleep debt than low intake group (OR = 1.41, *P* = 0.022).

**Conclusions:**

Children who had consumed sugar-sweetened beverages frequently slept for shorter durations at night on school days and exhibited greater sleep debt. The causal relationship was not clear. Nonetheless, these two factors are important in promoting children health.

## Introduction

Short sleep durations are associated with child obesity (including BMI, waist circumference, body fat, and body weight) (1–3). Short sleep durations may also influence children's blood sugar levels, blood pressure levels (3, 4), emotional management, academic performance, and quality of life (2). According to guidelines released by the National Sleep Foundation in 2015, children aged 6–13 years require 9–11 h of sleep per day, with their possibly acceptable hours of sleep ranging from 7 to 12 h (5). The American Academy of Sleep Medicine contended in 2016 that children aged 6–12 years must sleep for 9–12 h per day (6). These recommendations were aimed at broad public health monitoring and may, therefore, not be applicable to every individual; thus, because of interindividual variability, sleep duration has no ideal value (7). To overcome the interindividual variability, sleep debt measurement has been proposed in recent years (8–11). Studies have reported that sleep debt increases the risk of injury among children aged 6–12 years (8) and obesity among adolescents (9, 12), and it results in a drop in high density lipoprotein, a rise in insulin resistance (9) and depression, and inferior health (13) or academic performance (13). In summary, short sleep durations and sleep debt have considerable effects on the physical and psychological health of children and adolescents.

Diet plays a major role in the relationship between sleep and health (14). A system review combined 29 articles with subjects aged between 8 and 60 years old found that those consuming higher amount of processed and free-sugar food had worse sleep quality. However, the causal relationship was yet to be investigated (14). Other review studies have reported that most studies have regarded diet as a confounder in the relationship between sleep and cardiometabolic health, and some have regarded diet as a mediator or a modifier of said relationship (15). Sugar-sweetened beverages (SSBs), which are prevalent in children's diets, are a major public health concern; excessive SSBs consumption leads to obesity, diabetes, high uric acid, cardiovascular diseases, and low diet quality (16–18). Although the energy intakes from SSBs (not including sweetened tea or coffee) was decreasing in western countries, there were over 40% consumers aged between 2 and 19 years taking 0–20 oz SSBs (19). Research in China reported that adolescents who sleep for no more than 7 h consume a high amount of SSBs per day (20). However, few studies in Asia have addressed the relationship between children's sleep and SSBs. Similar to most Asian countries, sweetened tea is more frequently consumed than soda in Taiwan (18, 21). Accordingly, because of the differences between Eastern and Western societies in terms of culture, diet, and ethnicity, the relationship between SSBs and sleep may also differ between said societies.

Taiwan is one of the countries with the highest density of convenience stores and shops selling freshly made beverages in the world. By the end of 2017, 10,662 major convenience stores and 20,346 beverage shops had been established in Taiwan, representing one convenience store or beverage shop for approximately every 1,104 residents (22, 23). Such a high density has contributed to the high accessibility of SSBs to children and adolescents. Studies conducted between 2019 and 2020 have reported that adolescents consume SSBs as much as 413 g per day, and nearly every individual consumed SSBs at least once per week (18, 21). Therefore, the goal of this study was to preliminarily clarify elementary students' hours of nightly sleep and sleep debt, the proportion of the population with different sleep durations, and the correlation between SSBs consumed and sleep duration and sleep debt. It is hoped to provide more evidence for controlling SSBs intake in children.

## Methods

### Study Population

A cross-sectional study was conducted using data from two surveys of the Nutrition and Health Survey in Taiwan (NAHSIT) conducted in 2012 and in 2013~2016. The targets of analysis were elementary students aged between 6 and 12 years.

The NAHSIT were conducted regularly and employed stratified cluster sampling schemes from 1993. In the 2012 survey, elementary students aged 6~12 years or older were sampled. Specifically, Taiwan was divided into six regions according to population density, geographic location, and the degree of urbanization. With the probabilities proportional to each region's population size (PPS), four elementary schools were sampled from each region for a total of 24 schools; 50 students, with an equal ratio between male and female students and even distribution among the six grades, were sampled from each of the selected schools. A total of 1,200 students were surveyed, with a survey completion rate of 43.2%. In the survey conducted from 2013 to 2016, permanent residents aged 2 months or older were sampled. The 20 counties and cities in Taiwan were divided into 40 sample regions. Based on PPS, four townships were selected from each region for a total of 160 townships and 2,800 residents. The survey was divided into 4 years and yielded a completion rate of 74.2%. In both surveys, interviews and physical examinations were conducted, with written consent from their guardians; see related studies (18, 24) for further details.

### Measurement

#### Dietary Assessment

Data from the food frequency questionnaire (FFQ) was extracted to investigate the respondents' SSBs consumption frequency and breakfast regularity. The FFQ was validated in Taiwanese population with intraclass correlation coefficients for nutrient intakes 3 months apart ranged from 0.37 for saturated fat to 0.82 for alcohol (25). Regarding SSBs consumption frequency, the participants in the 2012 survey were asked “Did you have consumed soda, cola, milk tea or other sweetened beverages in the month?” and provided information on the frequency of consumption per day, per week, and per (25) month. In the 2013–2016 survey were asked “Did you have consumed, lactic acid beverages, carbonated beverages, sports beverages, energy beverages, chocolate beverages, milk tea, other tea beverages (with or without tea ingredients), or coffee in the month?” and provided information on the frequency of consumption per day, per week, and per month. The ratio of sugar-containing beverages each respondent consumed was adjusted according to selected frequency options. To improve data comparability, the medians in the data from the two surveys were designated as the cut-off points. Respondents were divided into two groups according to SSBs consumption frequency: the low intake group (<2 times/week in the 2012 survey and <4.7 times/week in the 2013–2016 survey) and the high intake group (≥2 times/week in the 2012 survey and ≥4.7 times/week in the 2013–2016 survey). Regarding breakfast regularity, the participants who had eaten breakfast every day were categorized in the regular group, and those who had eaten breakfast less than seven times per week comprised the irregular group.

#### Sleep Duration and Sleep Debt

Concerning sleep-related variables, the 2012 survey focused on the participants' average hours of nightly sleep in the 7 days preceding the survey, including school days and weekends. Slight adjustments were made to the sleep examined in 2013–2016 survey. First, the participants' sleep duration were recorded the participants' 3-day physical activity logs (3-d PALs) (26). Subsequently, the time participants went to bed and woke up during school days and weekends was recorded through the selection of a list of options, with intervals of 30 min. Finally, said time was recorded again through self-reports. The sleep variables, recorded in hours after organization, included the nightly sleep duration on school days and on weekends as well as sleep debt. Sleep debt was calculated by subtracting the sleep duration on school days from the sleep duration during weekends according to most studies (8–11). The cut-off points for the three aforementioned variables were quartile 1 (Q1) and quartile 3 (Q3), and each variable was divided into three groups: sleep duration on school days (<8.5 h,≥8.5 h and <9.5 h, and ≥9.5 h), sleep duration during weekends (<9 h, ≥9 h and <10.5 h, and ≥10.5 h), and sleep debt (0 h, >0 h and <2 h, and ≥2 h).

#### Covariates

Other variables from the NAHSIT questionnaires included age, sex, physical activity (PA), and family variables. BMI data, calculated as body weight (kg) divided by the square of body height in meters (m), were acquired through body measurements during the survey. 42.68% of the BMI data were missing in the 2013–2016 survey, the total percentage of missing BMI data of the two surveys was 27.55%. Therefore, the body weights and heights of the participants were interpolated 5 times using multiple imputation procedure in SUDAAN, using surveys, their age, sex, and PA as imputation data. The average of the five interpolated values was applied as the finalized representative value for recalculating the BMIs. Only one value could not be interpolated in the final results; thus, only one missing value was present in the BMI data.

Three versions of PA variables were implemented in the NAHSIT. The 2012 survey focused on the sports or activities the participants had frequently performed for at least 5 continuous minutes per week in the month prior to the survey as well as the weekly total activity duration (an open-ended question). The 2013–2016 survey examined 3-d PALs and their responses to the international physical activity questionnaire (IPAQ) (27). For analysis consistency, the PA data from the 2012 survey were converted to the IPAQ calculation model; the metabolic equivalents of tasks (METs) for vigorous PA, moderately PA, and walking, namely 8.0, 4.0, and 3.3, respectively, were multiplied by the number of days and durations of said PA, and the results were summed to obtain the weekly amount of PA (MET-mins/week) (28). The 3-d PAL data were converted to MET-mins/week by multiplying the daily METs of PA excluding sleeping, sitting, and standing by 60 minutes and 7 days (26). Because a larger variation was observed in the PA data from the 3-d PAL relative to those from the IPAQ, the median PA of each version of the questionnaire was used as the cut-off point, and respondents were divided into low and high PA groups. The median PA in IPAQ varied with different versions of questionnaire from 372 to 632, and the median PA of the 3-d PAL was 2234.4.

The family variables included parents' education level, employment status, and marital status. Education level was divided into junior high school and below, senior high school, and college and above; employment status was divided into employed (including part-time) and unemployed; marital status was divided into married (including cohabiting but unmarried) and others (e.g., divorced, separated, and widowed).

### Statistical Analyses

SAS version 9.4 (SAS Institute Inc., Cary, NC, USA) was employed to consolidate and debug the data and organize the variables. Because complex sampling scheme was used in the survey, SUDAAN version 11.0 was applied to estimate and analyse the data with sampling weights to represent the population in the two surveys. The difference between the high intake and low intake groups in continuous values was analyzed using a *t*-test. The categorical variables (e.g., demographic information, grouped sleep durations, and grouped sleep debt) were analyzed using a χ^2^ test. Changes in sleep duration and sleep debt across age groups were analyzed using a trend test and are illustrated using means and line graph. The distribution of sleep duration per hour and sleep debt per hour presented in percentages and line graph. The correlation of SSBs intake with sleep duration and sleep debt was analyzed using multinomial logistic regression, with model 1 (simple model) controlling for age and sex and model 2 (full model) controlling for all demographic variables.

### Sensitivity Analyses

Two sensitivity analyses were conducted: one on high and low-caffeine SSBs and the other on BMI data before and after imputation. Because caffeinated beverages maybe affect sleep duration and quality, the SSBs in this study were divided into caffeinated SSBs (e.g., sweetened coffee and tea) and other SSBs (e.g., low-caffeine or non-caffeinated SSBs). Each of the two types of SSBs was divided into two groups according to its median (the cut-off point). Before interpolation, 27.6% of the BMI in this study data were missing. To clarify whether imputation resulted in changes in the original data distribution and their significance, data in the BMI table before and after imputation were analyzed.

## Results

**Figure 1:**
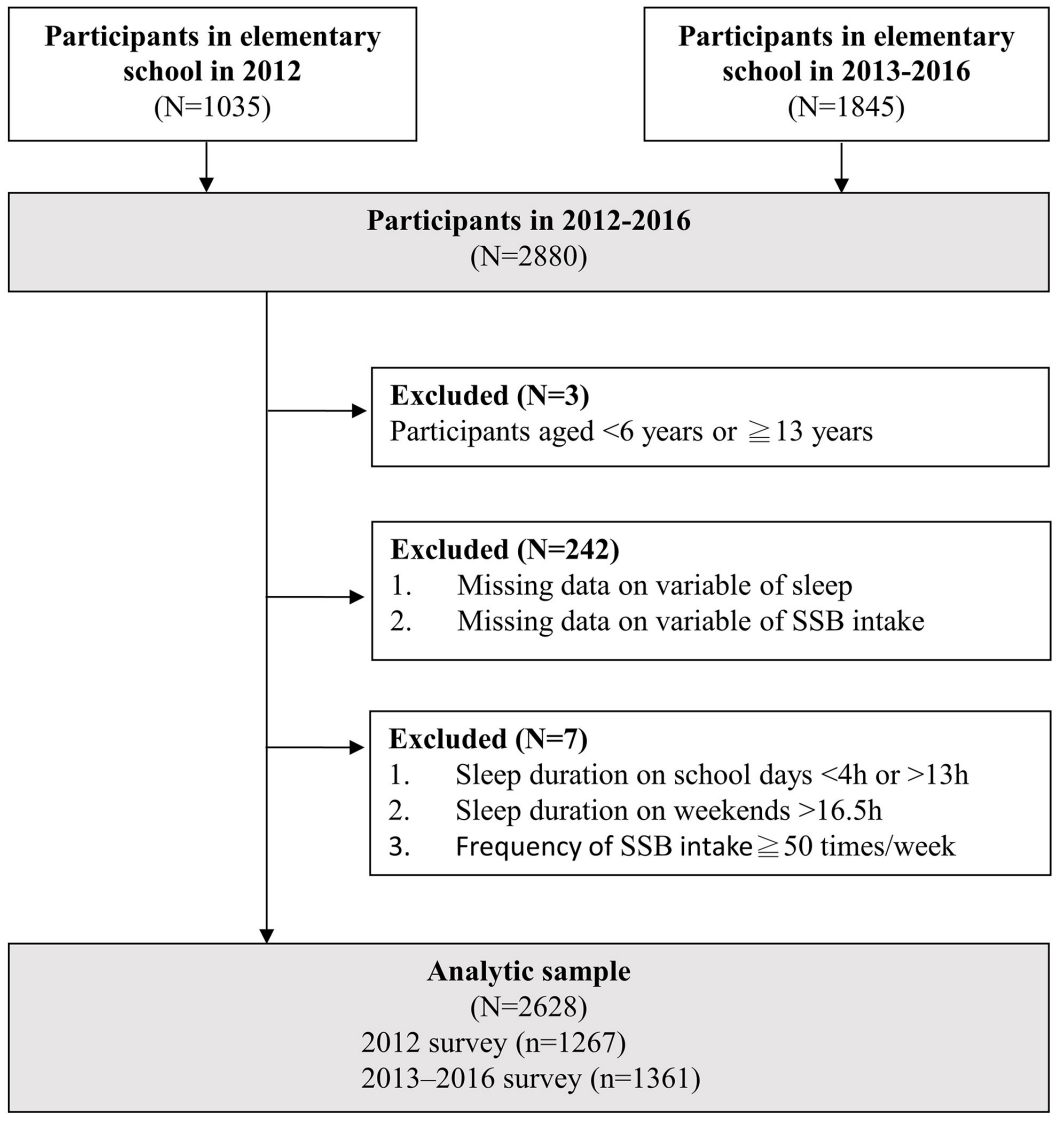
Flowchart of the study participants.

A total of 2,628 responses were analyzed (1,030 from the 2012 survey and 1,598 from the 2013–2016 survey, see Figure 1). Table 1 illustrates the demographic distribution of the surveyed elementary students aged 6–12 years. The average age was 9.4 years. The average age of the students in the high SSBs intake group was 0.3 years higher than that of those in the low SSBs intake group (*P* = 0.008).

**Table 1:** Characteristics of sampled children aged 6–12 years.

	**All (*****n*** **=** **2,628)**		**SSBs consumption**				
	***N***	**% (weight)**	**Low intake (*****n*** **=** **1,267)**		**High intake (n** **=** **1,361)**		***P-*value**
			***N***	**% (weight)**	***N***	**% (weight)**	
Age (y), mean (SE)	9.4 (0.1)		9.2 (0.1)		9.5 (0.2)		0.008
Age group (years)							0.001
6–9	1,338	49.9	706	54.7	632	45.1	
10–12	1,290	50.1	561	45.3	729	54.9	
Gender							0.678
Boys	1,323	52.2	594	51.4	729	52.9	
Girls	1,305	47.8	673	48.6	632	47.1	
BMI Categories^*^							0.581
Normal weight and Underweight	1,926	71.5	948	72.2	978	70.8	
Overweight and obesity	701	28.5	319	27.8	382	29.2	
PA (MET-minute/week)							0.246
Low	1,277	50.0	637	51.7	640	48.2	
High	1,293	50.0	601	48.3	692	51.8	
Breakfast eating (day/ week)							0.006
Not regular (<7)	374	15.0	135	10.6	239	19.3	
Regular (=7)	2,254	85.0	1132	89.4	1122	80.7	
Paternal education							0.002
Junior high school and below	487	17.5	176	13.4	311	21.5	
Senior high school	1,024	38.4	462	36.2	562	40.6	
College and above	1,060	44.1	602	50.4	458	37.8	
Maternal education							<0.001
Junior high school and below	463	14.8	176	10.7	287	18.9	
Senior high school	1,014	41.2	423	35.4	591	46.9	
College and above	1,056	44.0	624	53.9	432	34.2	
Paternal employed status							0.493
None	116	3.9	56	4.3	60	3.4	
Work (including part-time)	2,351	96.1	1142	95.7	1209	96.6	
Maternal employed status							0.347
None	594	26.2	299	25.0	295	27.5	
Work (including part-time)	1,868	73.8	908	75.0	960	72.5	
Parental marital status^†^							0.126
Married	2,138	84.2	1063	86.1	1075	82.3	
Other	460	15.8	185	13.9	275	17.7	

* *Multiple imputation*.

† *Marital status: married includes those living together but not married (cohabiting); other includes being divorced, separated, or widowed*.

**Figure 2:**
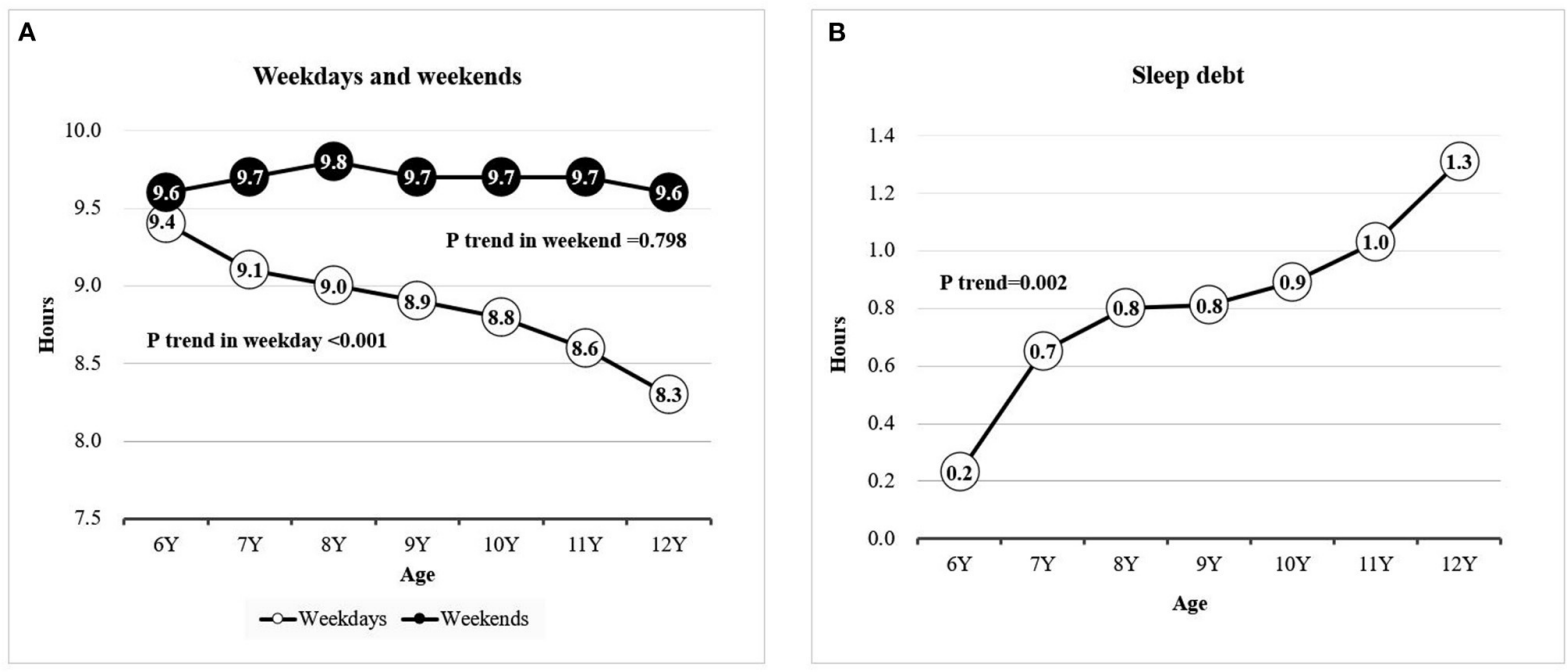
Mean sleep duration by age. **(A)** Sleep duration on weekdays and on weekends; **(B)** mean sleep debt.

Figure 2 showed the changes in the trend of average sleep duration and sleep debt across age. The average sleep duration on school days dropped slowly from 9.4 h in students aged 6 years to 8.3 h in those aged 12 years (*P* trend < 0.001); most of the students slept for ≥9.5 h during weekends, and no significant differences were noted across the age groups (Figure 2A). The difference between sleep duration on school days and during weekends was the smallest in the students aged 6 years, with the sleep debt being only 0.2 h; sleep debt was the largest in students aged 12 years (1.3 h; *P* trend = 0.002; Figure 2B).

**Figure 3:**
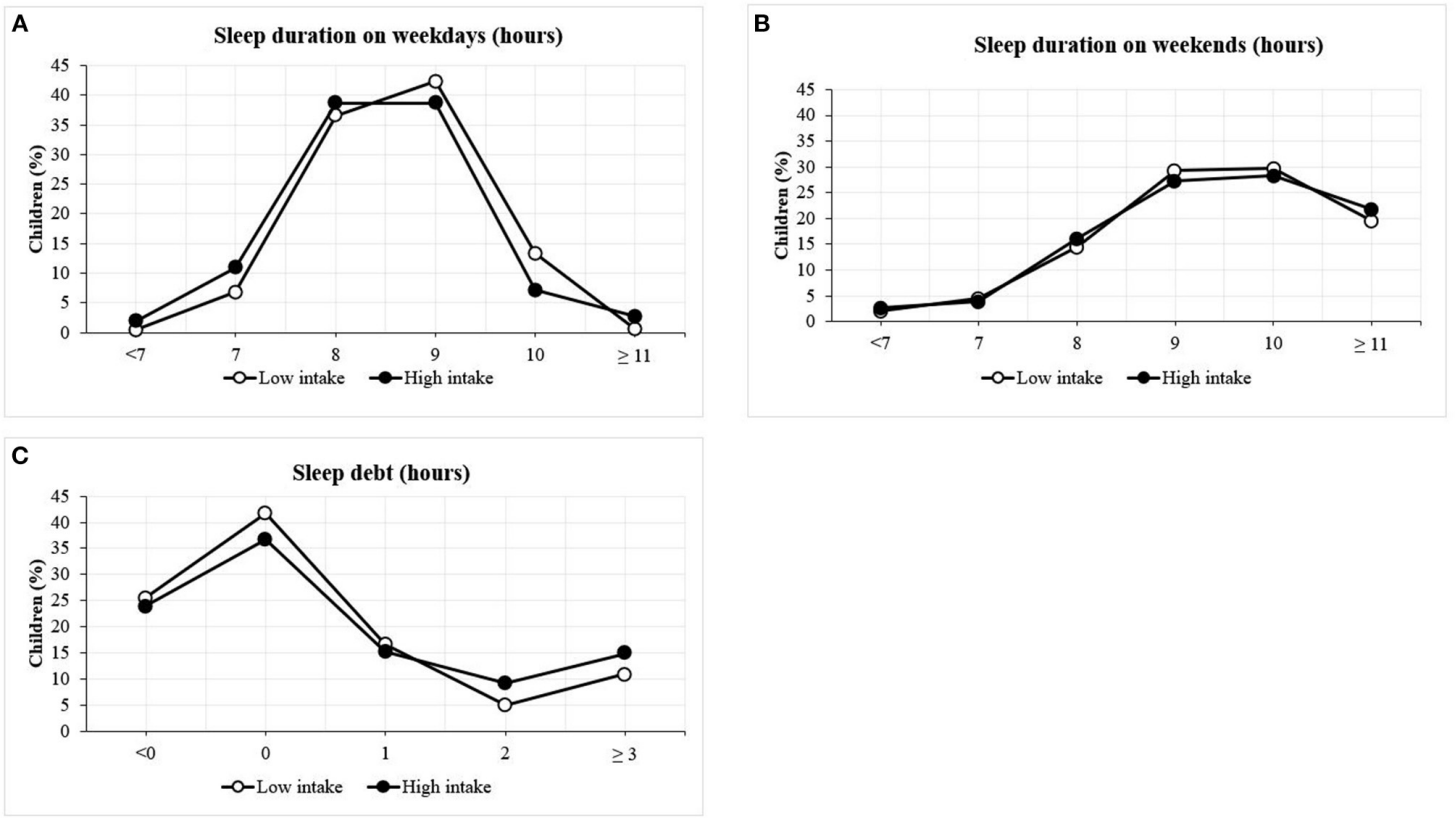
Distribution of sleep duration according to SSBs intake among children. **(A)** Sleep duration on weekdays; **(B)** sleep duration on weekends; **(C)** sleep debt.

Table 2 listed the means and population distribution of sleep duration and sleep debt in the high and low SSBs intake groups. No significant difference was observed between sleep duration during weekends and SSBs intake. Compared with the high intake group, the low intake group slept 0.2 h longer on school days (*P* < 0.001); 30% of the students in the low intake group slept for ≥9.5 h, which was 8% higher than those in the high intake group (*P* < 0.001); even though the average sleep debt in the low intake group was only 0.2 h lower than that in the high intake group, the level of significance was close to the threshold value (*P* = 0.049). The high intake group had a higher percentage of students who slept for <9 h on school days, whereas the low intake group had a higher percentage of those who slept for ≥9 h on school days (Figure 3A). The two groups were similar in the distribution of sleep duration per hour during weekends (Figure 3B). The low intake group had a higher prevalence of students whose sleep debt was <2 h, whereas the high intake group exhibited a higher prevalence of those with ≥2 h of sleep debt (Figure 3C).

**Table 2:** Distribution of participants by sleep pattern, including sleep duration on weekdays, sleep duration on weekends, and sleep debt, and SSBs consumption.

**Sleep duration and sleep debt**	**All (*****n*** **=** **2,628)**		**SSBs consumption**				
	***N***	**% (weight)**	**Low intake (*****n*** **=** **1,267)**		**High intake (n** **=** **1,361)**		***P-*value**
			***N***	**% (weight)**	***N***	**% (weight)**	
**Sleep duration**							
Weekdays (h), mean (SE)	8.8 (0.0)		8.9 (0.0)	8.7 (0.0)	<0.001
Weekdays in group							<0.001
< 8.5 h (Q1)	718	26.8	290	22.3	428	31.3	
≥ 8.5 h to < 9.5 h	1245	47.1	608	47.7	637	46.5	
≥ 9.5 h (Q3)	665	26.1	369	30.0	296	22.2	
Weekends (h), mean (SE)	9.7 (0.0)	9.7 (0.1)	9.7 (0.1)	0.722
Weekends in group							0.748
< 9 h (Q1)	560	22.0	252	21.2	308	22.7	
≥ 9 h to < 10.5h	1258	49.7	631	51.2	627	48.2	
≥ 10.5 h (Q3)	810	28.3	384	27.6	426	29.0	
**Sleep debt**							
Hours, mean (SE)	0.9 (0.1)		0.8 (0.1)	1.0 (0.1)	0.049
In group							0.058
≤ 0 h (Q1)	835	33.3	406	34.8	429	31.9	
> 0 h to < 2h	1101	40.9	569	42.9	532	39.0	
≥ 2h (Q3)	692	25.7	292	22.3	400	29.1	

Tables 3–5 presented the multinominal logistic regression analysis of the association of SSBs intake with sleep duration and sleep debt. A simple model for controlling age and sex and a full model for controlling all the demographic variables were presented (two analyses were conducted by two surveys: Supplementary 1–6). For sleep duration on school days (Table 3), relative to those in the low intake group, students in the high intake group were more likely to sleep <8.5 h than ≥9.5 h; the ORs of the simple and full models were, respectively, 1.72 and 1.57 (*P* < 0.05). The probability of the students in the high intake group sleeping for 8.5–9.5 h was also significantly higher than that of sleeping for ≥9.5 h; however, this was only noted in the simple model, with an OR of 1.27 (*P* = 0.04). No significant association was identified between SSBs intake and sleep duration during weekends (Table 4). Relative to those in the low intake group, the students in the high intake group exhibited a higher probability of sustaining ≥2 h of sleep debt than to have no sleep debt (Table 5).

**Table 3:** Multinomial logistic regression analysis for the sleep duration on weekdays.

SSBs^*^	** <8.5 hours**			**≥8.5 to** ** <9.5 h**			**≥9.5 h**
	**OR**	**(95% CI)**	***P-*value**	**OR**	**(95% CI)**	***P-*value**	**OR**
**Model 1 (simple model)^†^**
Low intake	1.00			1.00			1.00
High intake	1.72	(1.37, 2.15)	<0.001	1.27	(1.01, 1.58)	0.040	1.00
**Model 2 (full model)^†^**
Low intake	1.00			1.00			1.00
High intake	1.67	(1.23, 2.26)	0.002	1.08	(0.83, 1.41)	0.552	1.00

* *Model 1 adjusted for survey, age, gender; model 2 adjusted for age, gender, BMI categories, breakfast eating, physical activity, paternal education, maternal education, parental marital status, paternal employed status, and maternal employed status*.

† *Respondents were divided into two groups according to SSBs consumption frequency. The low intake group (<2 times/week in the 2012 survey and <4.7 times/week in the 2013–2016 survey) and the high intake group (≥ 2 times/week in the 2012 survey and ≥ 4.7 times/week in the 2013-2016 survey)*.

**Table 4:** Multinomial logistic regression analysis for the sleep duration on weekends.

SSBs^*^	** <9 h**			**≥9 to** ** <10.5 h**			**≥10.5 h**
	**OR**	**(95% CI)**	***P-*value**	**OR**	**(95% CI)**	***P*-value**	**OR**
**Model 1 (simple model)^†^**
Low intake	1.00			1.00			1.00
High intake	0.98	(0.74, 1.29)	0.878	0.88	(0.63, 1.21)	0.405	1.00
**Model 2 (full model)^†^**
Low intake	1.00			1.00			1.00
High intake	0.97	(0.66, 1.43)	0.888	0.90	(0.63, 1.27)	0.518	1.00

* *Model 1 adjusted for survey, age, gender; model 2 adjusted for age, gender, BMI categories, breakfast eating, physical activity, paternal education, maternal education, parental marital status, paternal employed status, and maternal employed status*.

† *Respondents were divided into two groups according to SSBs consumption frequency. The low intake group (<2 times/week in the 2012 survey and <4.7 times/week in the 2013–2016 survey) and the high intake group (≥2 times/week in the 2012 survey and ≥4.7 times/week in the 2013–2016 survey)*.

**Table 5:** Multinomial logistic regression analysis for the sleep debt.

SSBs^*^	**≥2 h**			**>0 to** ** <2 h**			**0 h (No sleep dept)**
	**OR**	**(95% CI)**	***P* value**	**OR**	**(95% CI)**	***P* value**	**OR**
**Model 1 (simple model)^†^**
Low intake	1.00			1.00			1.00
High intake	1.38	(1.04, 1.83)	0.029	0.97	(0.75, 1.25)	0.806	1.00
**Model 2 (full model)^†^**
Low intake	1.00			1.00			1.00
High intake	1.41	(1.06, 1.88)	0.022	1.13	(0.85, 1.5)	0.386	1.00

* *Model 1 adjusted for survey, age, gender; model 2 adjusted for age, gender, BMI categories, breakfast eating, physical activity, paternal education, maternal education, parental marital status, paternal employed status, and maternal employed status*.

† *Respondents were divided into two groups according to SSBs consumption frequency. The low intake group (<2 times/week in the 2012 survey and <4.7 times/week in the 2013–2016 survey) and the high intake group (≥2 times/week in the 2012 survey and ≥4.7 times/week in the 2013–2016 survey)*.

Two sensitivity analyses results were conducted including the analyse of high and low-caffeine SSBs and the analyse of BMI data before and after imputation. First, after the confounding factors were controlled and the data were analyzed through multinomial logistic regression, slight but non-significant changes were observed in the odds ratios (ORs) of high and low-caffeine SSBs. That is, sleep durations and sleep debt were not correlated with the caffeine contents of SSBs. The results related to the caffeine content of SSBs are not presented in this paper. Second, the original data distribution before BMI imputation was compared with after imputation. Among all the demographic variables, the difference between BMI categories exhibited only 4% differences. Multinomial logistic regression analysis results indicated slight but non-significant changes in some of the ORs (≤0.2). Therefore, the imputed BMI values were included in the final analysis.

## Discussion

The current results indicated that the students' sleep duration on school days decreased slowly over age and their sleep debt increased. Moreover, compared with the low intake group, the high intake group exhibited a higher prevalence of sleeping <8.5 h on school days and a lower prevalence of sleeping for ≥9.5 h. After the confounding factors were controlled, the high intake group exhibited a higher probability of sleeping for <8.5 h on school days and sustaining ≥2 h of sleep debt than the low intake group; however, no significant difference was identified between the two groups for sleep duration during weekends.

In Taiwan, elementary students are provided with at least 30 min for an afternoon nap as part of their daily activities, but the exact length varies by school; this activity pattern is similar to that in China (29). This study only focused on sleep duration at night; however, when the afternoon nap is included, elementary students' sleep duration on both school days and weekends satisfies the 9-h recommendation of the National Sleep Foundation and the American Academy of Sleep Medicine. Moreover, the total sleep duration on school days in the current study is close to the average sleep duration on school days as reported in systematic review studies on children aged 6–12 years (9.2 h of sleep per 24 h) (30). According to the standard values proposed in a 2019 systematic review on deficient sleep in people of various age groups, (31) students aged 6 years slept for slightly shorter than the recommended duration of 9.5 h, but those in other age groups satisfied said standard.

According to a systematic review of studies that employed wrist actigraphy to measure nightly sleep duration, children aged 9–11 years sleep for 8.7 and 8.8 h on school days and weekends, respectively (32); because the two values do not differ considerably, nearly no sleep debt was identified. Comparatively, children in Taiwan sleep longer on weekends, resulting in greater sleep debt (0.9 h). Children's sleep duration at night on school days decreased as they aged, whereas their sleep duration at night during weekends did not change significantly as they aged; consequently, their sleep debt increased as they became older. Similar results have been reported in relevant research on children and adolescents aged 11–15 years in other countries (11).

This study reported that frequent SSBs consumption in children is significantly associated with lower sleep duration at night on school days and greater sleep debt; this is consistent with the findings in other studies (33). A Canadian study showed that middle school (aged 11–14 years) students with shorter sleep had higher chance of consuming SSBs (OR = 1.64, *P* = 0.002), whereas the association did not appear in high school students (33). In a study on children aged 8–11 years in Denmark, SSBs energy intake density (energy intake / body weight × 100%) was reported to be negatively correlated with sleep duration at night (β = −1.07, *P* < 0.001) (34). A study focusing on 12 countries revealed that the more frequently children aged 9–11 years consume soft beverages (e.g., cola), the shorter their sleeping duration at night (*P* trend < 0.01) (35). According to a systematic review on children aged 2–19 years, short sleep duration is positively correlated with soda consumption [OR = 1.16, 95% confidence interval (CI): 1.09–1.25] (36). Concerning studies in Asia, research in China reported that children and adolescents aged 6–17 years who sleep for <7 h per day consume a high quantity of SSBs per day (OR = 1.29, 95% CI: 1.19–1.40) (20). Similarly, a study in Iran indicated that children and adolescents who sleep for short durations exhibit a particularly high probability of consuming soft beverages and tea (both sweetened and unsweetened) (*P* < 0.05) (37).

Few studies have focused on the association between children's sleep debt and SSBs intake. Studies on adults aged 18–39 years have indicated that perceived sleep debt is positively associated with the daily amount of SSBs consumption (*P* = 0.02) (38). According to a study on children aged 8–17 years, those with shorter sleep durations on school days (*P* = 0.01) and longer weekend catch-up sleep (i.e., sleep debt; *P* = 0.004) tend to be eating in the absence of hunger (39). Adolescents aged 15 years with higher social jetlag (i.e., the absolute values of sleep debt) are more likely to consume two or more servings of SSBs per day (OR = 1.21, *P* < 0.001) (40). Sleep pressure is modulated by homeostatic and circadian process) (41). Sleep plasticity is a complex neurobiological system. It is important to human immunity, cognition, and energy storage. Insufficient sleep increases sleep pressure, thus results in a lot of sleep debt. This might affect behavior and cognitive functions of children (41, 42).

The causal relationship between short sleep duration and SSBs consumption has yet to be clarified. One study on the dietary reactions of people deprived of sleep involved a 14-day experiment with 16 healthy men. When they obtained sufficient sleep, the participants exhibited a lower sense of hunger before meals and higher sense of satiety after meals; when completely deprived of sleep, the participants' brains became hyperreactive to hints of food, which heightened their food craving (43). Another study showed thar the participants exhibited 6% higher mean daily dietary energy intake after sleeping for only 5 h per day than they did after sleeping for 9 h per day (*P* < 0.05); in addition, they exhibited higher weights (*P* < 0.05), higher carbohydrate intake (*P* < 0.001), lower breakfast intake, and higher post dinner snack intake (*P* < 0.05) after sleeping for only 5 h per day. Said post dinner snacks primarily consisted of carbohydrates (*P* = 0.001), protein (*P* < 0.001), and fiber (*P* < 0.001) (44). As reported in a systematic review, (36) people with short sleep durations tend to consume low quantities of fruits and vegetables and high quantities of snacks and soda. Therefore, in addition to starch and high-sugar snacks, increases in carbohydrate intake can be attributed to SSBs consumption. In Taiwan, because of the prevalence of convenience stores and beverage shops and the low prices of SSBs, SSBs are highly accessible; this increases the probability of SSB overconsumption by children.

This study focused on the association between SSBs consumption and children's sleep, which has rarely been explored. When children's behavior on school days and weekends change, the weighted average sleep duration in a single week does not reflect their actual sleep status. This study separated sleep duration on school days from those on weekends. The sleep debt highlights sleep differences within individuals; this is an area rarely explored by studies on children. Furthermore, this study provided supporting information on the differences between Asian and Western societies concerning the primary types of SSBs consumed, which have also rarely been examined in research. However, this study has some limitations. First, because this was a cross-sectional study, it could not identify a causal relationship between SSBs consumption and sleep. Whether SSBs overconsumption affects children's sleep durations or sleep durations affect children's SSBs consumption frequency requires further clarification. Second, because a wide range of SSBs are typically consumed, the effect of each type of SSBs on sleep durations is difficult to examine. Therefore, this study employed sensitivity analysis on the correlation between the caffeine content in SSBs (including sweetened coffee and tea) and sleep duration. The results indicated that the caffeine content in SSBs was not significantly associated with sleep duration. Third the data acquired in this study were based on the food frequency questionnaire, which only provided information on SSBs consumption frequency rather than the actual quantity consumed, which might be major factor in sleep duration. Nevertheless, SSBs consumption frequency analysis more favorably reflects children's long-term beverage consumption behavior and sleeping habits and provides a reference for policy-making. Finally, this was a secondary data analysis from a national nutritional survey, which included people in all age. The questions on sleep were self-reported. It was difficult to incorporate the full pediatric sleep assessment scales not to mention the use instrument evaluation in a large population survey. However, the self-reported questions on sleep and FFQ could still identify the problem and provide useful information for related prevention.

## Conclusion

This study revealed that children aged 6–12 years who consume SSBs more frequently are prone to exhibit shorter sleep durations and greater sleep debt. Both children who consume SSBs frequently and those who exhibit sleep problems are prone to exhibit physiological, psychological, and behavioral problems, such as being overweight, having high blood sugar or depression, or demonstrating poor academic performance. The reduction of SSBs availability and consumption frequency must be prioritized in solving these problems. Coordination and cooperation between parents and children are required for the management and adjustment of children's sleep duration on school days. For example, sleep duration at night can first be extended by 10–30 min and further lengthened afterwards to increase children's sleep duration and eliminate their sleep debt. Therefore, the Taiwanese government should prioritize improving people's childhood eating habits and behavior when formulating and promoting policies related to daily living.

## Data Availability Statement

The raw data supporting the conclusions of this article will be made available by the authors, without undue reservation.

## Ethics Statement

This study was approved by the Institutional Review Board (IRB) of the National Health Research Institutes (approval No: EC1090206-E). Written informed consent was obtained from the minor(s)' legal guardian/next of kin for the publication of any potentially identifiable images or data included in this article.

## Author Contributions

Y-HS undertook data analysis and interpretation and wrote the manuscript. H-CW provided a critical review of the manuscript. W-HP was responsible for the nutritional surveys and provided a critical review of the manuscript. H-YC provided the conceptual framework of this study, supervised the analysis, and revised the manuscript. All authors read and approved the final manuscript.

## Funding

This study was supported by the National Health Research Institutes. The content of this paper reflects only the authors' views. The sponsors did not place any restrictions on the study design. This work was funded by the Health Promotion Administration, Ministry of Health and Welfare (MOHW110-HPA-H-114-144703). The content of this research may not represent the opinion of the Health Promotion Administration, Ministry of Health and Welfare.

## Author Disclaimer

The views expressed herein are solely those of the authors.

## Conflict of Interest

The authors declare that the research was conducted in the absence of any commercial or financial relationships that could be construed as a potential conflict of interest.

## Publisher's Note

All claims expressed in this article are solely those of the authors and do not necessarily represent those of their affiliated organizations, or those of the publisher, the editors and the reviewers. Any product that may be evaluated in this article, or claim that may be made by its manufacturer, is not guaranteed or endorsed by the publisher.
