# Supplementary material for: The Association Between Frequent Sugar-Sweetened Beverage Intake and Sleep Duration in School Children: A Cross-Sectional Study
Source: Front Nutr. 2022 Mar 15;9:847704. doi: 10.3389/fnut.2022.847704 (PMC8965345; doi:10.3389/fnut.2022.847704)
Supplement: Supplementary file 1 [file Table_1.DOCX]

Supplementary 1. Multinomial logistic regression analysis for the sleep duration on weekdays in 2012.

| **SSBs** | **< 8.5 hours** | | |  | **≥ 8.5 to < 9.5 hours** | | |  | **≥ 9.5 hours** |
| --- | --- | --- | --- | --- | --- | --- | --- | --- | --- |
|  | **OR** | **(95% CI)** | ***P* value** |  | **OR** | **(95% CI)** | ***P* value** |  | **OR** |
| **Model 1 (simple model)** |  |  |  |  |  |  |  |  |  |
| **Low intake** | 1.00 |  |  |  | 1.00 |  |  |  | 1.00 |
| **High intake** | 1.85 | (1.48, 2.31) | <0.001 |  | 1.39 | (1.07, 1.80) | 0.016 |  | 1.00 |
| **Model 2 (full model)** |  |  |  |  |  |  |  |  |  |
| **Low intake** | 1.00 |  |  |  | 1.00 |  |  |  | 1.00 |
| **High intake** | 1.74 | (1.27, 2.36) | 0.001 |  | 1.07 | (0.78, 1.47) | 0.455 |  | 1.00 |

Supplementary 2. Multinomial logistic regression analysis for the sleep duration on weekends in 2012.

| **SSBs** | **< 9 hours** | | |  | **≥ 9 to < 10.5 hours** | | |  | **≥ 10.5 hours** |
| --- | --- | --- | --- | --- | --- | --- | --- | --- | --- |
|  | **OR** | **(95% CI)** | ***P* value** |  | **OR** | **(95% CI)** | ***P* value** |  | **OR** |
| **Model 1 (simple model)** |  |  |  |  |  |  |  |  |  |
| **Low intake** | 1.00 |  |  |  | 1.00 |  |  |  | 1.00 |
| **High intake** | 1.04 | (0.70, 1.56) | 0.839 |  | 0.90 | (0.55, 1.48) | 0.669 |  | 1.00 |
| **Model 2 (full model)** |  |  |  |  |  |  |  |  |  |
| **Low intake** | 1.00 |  |  |  | 1.00 |  |  |  | 1.00 |
| **High intake** | 1.00 | (0.57, 1.76) | 0.991 |  | 0.91 | (0.53, 1.57) | 0.723 |  | 1.00 |

Supplementary 3. Multinomial logistic regression analysis for the sleep debt in 2012.

| **SSBs** | **≥ 2 hours** | | |  | **> 0 to < 2 hours** | | |  | **0 hour**  **(No sleep dept)** |
| --- | --- | --- | --- | --- | --- | --- | --- | --- | --- |
|  | **OR** | **(95% CI)** | ***P* value** |  | **OR** | **(95% CI)** | ***P* value** |  | **OR** |
| **Model 1 (simple model)** |  |  |  |  |  |  |  |  |  |
| **Low intake** | 1.00 |  |  |  | 1.00 |  |  |  | 1.00 |
| **High intake** | 1.32 | (0.86, 2.01) | 0.192 |  | 1.03 | (0.73, 1.46) | 0.860 |  | 1.00 |
| **Model 2 (full model)** |  |  |  |  |  |  |  |  |  |
| **Low intake** | 1.00 |  |  |  | 1.00 |  |  |  | 1.00 |
| **High intake** | 1.30 | (0.85, 2.01) | 0.215 |  | 1.21 | (0.82, 1.77) | 0.325 |  | 1.00 |

Supplementary 4. Multinomial logistic regression analysis for the sleep duration on weekdays in 2013-2016.

| **SSBs** | **< 8.5 hours** | | |  | **≥ 8.5 to < 9.5 hours** | | |  | **≥ 9.5 hours** |
| --- | --- | --- | --- | --- | --- | --- | --- | --- | --- |
|  | **OR** | **(95% CI)** | ***P* value** |  | **OR** | **(95% CI)** | ***P* value** |  | **OR** |
| **Model 1 (simple model)** |  |  |  |  |  |  |  |  |  |
| **Low intake** | 1.00 |  |  |  | 1.00 |  |  |  | 1.00 |
| **High intake** | 1.50 | (0.94, 2.39) | 0.084 |  | 1.09 | (0.91, 1.69) | 0.673 |  | 1.00 |
| **Model 2 (full model)** |  |  |  |  |  |  |  |  |  |
| **Low intake** | 1.00 |  |  |  | 1.00 |  |  |  | 1.00 |
| **High intake** | 1.63 | (0.93, 2.86) | 0.003 |  | 1.04 | (0.64, 1.69) | 0.876 |  | 1.00 |

Supplementary 5. Multinomial logistic regression analysis for the sleep duration on weekends in 2013-2016.

| **SSBs** | **< 8.5 hours** | | |  | **≥ 8.5 to < 10.5 hours** | | |  | **≥ 10.5 hours** |
| --- | --- | --- | --- | --- | --- | --- | --- | --- | --- |
|  | **OR** | **(95% CI)** | ***P* value** |  | **OR** | **(95% CI)** | ***P* value** |  | **OR** |
| **Model 1 (simple model)** |  |  |  |  |  |  |  |  |  |
| **Low intake** | 1.00 |  |  |  | 1.00 |  |  |  | 1.00 |
| **High intake** | 0.90 | (0.64, 1.28) | 0.797 |  | 0.84 | (0.60, 1.18) | 0.303 |  | 1.00 |
| **Model 2 (full model)** |  |  |  |  |  |  |  |  |  |
| **Low intake** | 1.00 |  |  |  | 1.00 |  |  |  | 1.00 |
| **High intake** | 0.96 | (0.74, 1.99) | 0.846 |  | 0.88 | (0.63, 1.24) | 0.458 |  | 1.00 |

Supplementary 6. Multinomial logistic regression analysis for the sleep debt in 2013-2016.

| **SSBs** | **≥ 2 hours** | | |  | **> 0 to < 2 hours** | | |  | **0 hour**  **(No sleep dept)** |
| --- | --- | --- | --- | --- | --- | --- | --- | --- | --- |
|  | **OR** | **(95% CI)** | ***P* value** |  | **OR** | **(95% CI)** | ***P* value** |  | **OR** |
| **Model 1 (simple model)** |  |  |  |  |  |  |  |  |  |
| **Low intake** | 1.00 |  |  |  | 1.00 |  |  |  | 1.00 |
| **High intake** | 1.46 | (1.04, 2.04) | 0.029 |  | 0.89 | (0.64, 1.23) | 0.470 |  | 1.00 |
| **Model 2 (full model)** |  |  |  |  |  |  |  |  |  |
| **Low intake** | 1.00 |  |  |  | 1.00 |  |  |  | 1.00 |
| **High intake** | 1.50 | (1.01, 2.22) | 0.045 |  | 1.00 | (0.69, 1.45) | 0.995 |  | 1.00 |
